# Supplementary material for: Getting More Out of Biomedical Documents with GATE's Full Lifecycle Open Source Text Analytics
Source: PLoS Comput Biol. 2013 Feb 7;9(2):e1002854. doi: 10.1371/journal.pcbi.1002854 (PMC3567135; doi:10.1371/journal.pcbi.1002854)
Supplement: Dataset S1 — GATE software. Dataset S1 bundled with this paper contains a distribution of GATE (or see http://gate.ac.uk/download/). (TGZ) [file pcbi.1002854.s001.tgz › plos-gate/plugins/Tagger_MetaMap/doc/gate/metamap/package-summary.html]

gate.metamap


---


|  |  |  |
| --- | --- | --- |
| **Overview** \| **Package** \| Class \| **Use** \| **Tree** \| **Deprecated** \| **Index** \| **Help** | |  |
| PREV PACKAGE   **NEXT PACKAGE** | **FRAMES**    **NO FRAMES**     **All Classes** |  |


---

## Package gate.metamap

| **Class Summary** | |
| --- | --- |
| **MetaMapPR** | This class is the implementation of the resource METAMAP. |

| **Enum Summary** | |
| --- | --- |
| **OutputMode** |  |
| **TaggerMode** |  |

---


|  |  |  |
| --- | --- | --- |
| **Overview** \| **Package** \| Class \| **Use** \| **Tree** \| **Deprecated** \| **Index** \| **Help** | |  |
| PREV PACKAGE   **NEXT PACKAGE** | **FRAMES**    **NO FRAMES**     **All Classes** |  |


---
